# Executive functional deficits during electrical stimulation of the right frontal aslant tract

**DOI:** 10.1007/s11682-020-00439-8

**Published:** 2021-01-19

**Authors:** Geert-Jan M. Rutten, Maud J. F. Landers, Wouter De Baene, Tessa Meijerink, Stephanie van der Hek, Jeroen H. B. Verheul

**Affiliations:** 1 Department of Neurosurgery, Elisabeth-Tweesteden Hospital Tilburg, Tilburg, The Netherlands; 2 Department of Cognitive Neuropsychology, Tilburg University, Tilburg, The Netherlands; 3 Department of Medical Psychology, Elisabeth-Tweesteden Hospital Tilburg, Tilburg, The Netherlands

**Keywords:** Frontal aslant tract, Direct electrical stimulation, Glioma, Executive functions, Awake surgery

## Abstract

Direct electrical stimulation mapping was used to map executive functions during awake surgery of a patient with a right frontal low-grade glioma. We specifically targeted the frontal aslant tract, as this pathway had been infiltrated by the tumor. The right frontal aslant tract has been implicated in executive functions in the neuroscientific literature, but is yet of unknown relevance for clinical practice. Guided by tractography, electrical stimulation of the frontal aslant tract disrupted working memory and inhibitory functions. In this report we illustrate the dilemmas that neurosurgeons face when balancing maximal tumor resection against optimal cognitive performance. In particular, we emphasize that intraoperative tasks that target cognitive functions should be carefully introduced in clinical practice to prevent clinically irrelevant responses and too early termination of the resection.

**Supplementary Information:**

The online version contains supplementary material available at 10.1007/s11682-020-00439-8.

## Introduction

Direct electrical stimulation (DES) has been widely accepted by neurosurgeons as a reliable tool to map sensorimotor and language functions during surgery. It is used intraoperatively to establish functional tumor boundaries and safely maximize tumor resection. Over the last years, there is accumulating evidence that many glioma patients are cognitively impaired, both before and after surgery, and that these impairments often disrupt an enjoyable and productive socioprofessional life (Rijnen et al. 2019). In response, several neurosurgical teams have introduced tasks during awake surgery to monitor cognitive functions (Motomura et al. 2020; Puglisi et al. 2019). There is, however, a potential danger to such an exploratory approach. Not all electrically excitable structures have similar functional relevance, and these new tasks may falsely indicate that a particular area or pathway is indispensable for certain cognitive performance. Mandonnet et al. recently proposed a roadmap to minimize these false-positive findings (Mandonnet et al. 2020). We agree that there should at least be a specific hypothesis about the functionality of the structure or region that is tested, preferably based on scientific or clinical knowledge. In this paper we illustrate how we got to testing of the right frontal aslant tract (FAT) during awake surgery in a patient with a glioma.

The FAT is a relatively newly described fiber pathway that connects the posterior parts of the inferior and superior frontal gyrus (Aron et al. 2007; Lawes et al. 2008). There is considerable evidence that the FAT in the left, language-dominant hemisphere is associated with motor speech functions (Dick et al. 2019; Kinoshita et al. 2015). The right FAT has been associated with executive functions (Aron et al. 2007; Varriano et al. 2018).

Recently, we were presented with a patient with a right frontal glioma whereby the tumor had partly infiltrated the FAT. It was decided to perform surgery under awake conditions to specifically test whether or not electrical stimulation of the FAT can indeed disrupt cognitive functions. In this paper we describe the clinical dilemmas we faced, as well as the results of the procedure.

## Methods and results

### Clinical case

Patient is a right-handed 42-year-old male that presented with a seizure. An MRI-scan revealed an intra-axial non-enhancing lesion in the right prefrontal lobe, typical for a low-grade glioma. The patient had no relevant medical history and upon neurological examination no abnormalities were found. His education level was intermediate vocational. He stated that he was doing well psychosocially, and he and his wife had not noticed any major changes in their personal life. Patient functioned adequately as a husband, as father of two small children, and as chief of his own small company.

### Preoperative cognitive testing

Neuropsychological assessment was performed one week before surgery as part of standard neurooncological care, and indicated impairments in phonemic fluency, motor speed, inhibition, verbal memory and shifting attention (see Table 1B) (Rijnen et al. 2017).

**Table 1:** Results from intraoperative stimulation mapping and pre- and postoperative neuropsychological test results

1A: Results from intraoperative stimulation mapping				
TAG NR	**LOCATION (based on anatomy and tractographic results)**	**TASK**	**STIMULATION SETTINGS** **mA / ms**	**OBSERVATIONS during intraoperative mapping (number or errors / total number of stimulations)**
1	precentral cortex	none	3 / 500	1/1 flexion left elbow
2	precentral cortex	none	3 / 500	1/1 movement left upper arm
3	premotor cortex	continuous flexion-extension movements of elbow, wrist, and hand	3 / 1000	1/1 movements of the hand were “blocked”. When asked what happened, patient said he was in doubt whether to continue or not
4	premotor cortex, termination of FAT	picture naming^1^	4 / 1000	3/5 hesitation; paraphasia
4	premotor cortex, termination of FAT	stroop^2^	4 / 1000	2/2 hesitation; speech arrest
5	FAT	stroop^2^	6 / 1000	6/10
5	FAT	digit span backward^3^	6 / 1000	8/9
6	FAT	digit span backward^3^	6 / 1000	3/3
6	FAT	digit span backward^3^	3 / 1000	3/4
7	FAT	digit span backward^3^	6 / 1000	7/9
7	FAT	digit span forward^3^	6 / 1000	2/9
8	FAT	digit span backward^3^	6 / 1000	2/2
1B: Pre- and postoperative neuropsychological test results				
NEUROPSYCOLOGICAL TASK			**PREOPERATIVE**^**4**^	**POSTOPERATIVE (corrected for practice effects)**^**4, 5**^
	CNS VS^6^	Verbal memory	Z −2,83, perc <1	Z −2,76, perc <1
		Visual memory	Z −1,41, perc 8	Z −0,11, perc 46
		Reaction time	Z −1,23, perc 12	Z −1,58, perc 6
		Motor speed	Z −8,21, perc <1	Z −1,18, perc 14
		Inhibition (stroop)	Z −4,04, perc <1	Z −0,94, perc 18 *
		Shifting attention	Z −3,70, perc. <1	Z −0,99, perc 17 *
		Processing speed	Z −1,19, perc 12	Z −2,40, perc 1
	Phonemic word fluency^7^		Z −2,0 perc 2	Z −0,9, perc 18 *
	Digit span forward^3^		Z −0,5, perc 31	Z −0,6, perc 26
	Digit span backward^3^		Z −0,6, perc 26	Z −0,4, perc 34

*meaning significant difference of *p* < .05 between pre- and postoperative

^1^Snodgrass and Vanderwart (1980)

^2^Delis et al. (2001)

^3^Wechsler (1987)

^4^Rijnen et al. (2017)

^5^Rijnen et al. (2018)

^6^Gualtieri and Johnson (2006)

^7^Schmand et al. (2008)

### Diffusion-weighted imaging and tractography

A standard presurgical imaging protocol was performed, that included functional MRI (resting state, motor and language task) and a diffusion-weighted MRI for tractography. A single-shell diffusion scheme was acquired (b= 1500s/mm^2^, 50 directions, 7 b=0 images, 2 mm isotropic voxel size), using a Philips Achieva 3 T MRI-scanner. Tractography was performed with Diffusion Tensor Imaging (DTI) and corticospinal tract, inferior fronto-occipital fasciculus and FAT were uploaded to the surgical guidance system for use during surgery (Stealth Viz software and Stealth S8, Medtronic). An immediate postoperative DTI scan was made for verification of positive and negative mapping sites.

### Shared decision making

We discussed with the patient and his wife, in a usual manner, the presumed diagnosis of a low-grade glioma and treatment possibilities. In particular, pros and cons of resective surgery were discussed in light of an optimal onco-functional balance. The dilemma we faced was whether or not we should aim for a total resection (according to FLAIR boundaries) given the fact that tractography indicated infiltration of the FAT by the tumor in the mesial frontal region. From a classic neurological point of view, this right frontal tumor was not bordering eloquent structures, making it a rather clear-cut case. There is no convincing evidence in the literature (yet) that damage to the right FAT results in permanent deficits. In our patient, cognitive tests already indicated impairments in several cognitive domains prior to surgery, suggesting that peritumoral regions were somehow implicated in cognitive functions and that the plastic potential of the brain had reached its limits. We added to that the results of our own recent retrospective patient study [results not yet published], that clearly suggested a role for the FAT in executive functions in frontal glioma patients. We discussed this dilemma openly with the patient and he gave informed consent to perform the surgery awake and specifically test top-down executive functions of the FAT. If indeed electrical stimulation of the FAT should result in reproducible errors during testing of a cognitive task, we planned to leave the FAT-part of the tumor behind.

### Intraoperative procedure

Awake surgery was performed using a combination of local anesthesia and sedation without mechanical ventilation (Arzoine et al. 2020; Rutten 2015). Electrical stimulation was performed with a bipolar probe and a biphasic 60 Hz current (Nim Eclipse, Medtronic USA). The patient was monitored during surgery by the same clinical neuropsychologist that had investigated him prior to surgery. Intraoperative tests had been practiced preoperatively, and performance was sufficient on picture naming, recall and working memory. Patient was significantly impaired on a set-shifting task preoperatively, that we therefore decided not to use during surgery.

Results from electrical stimulation are summarized in Table 1A. Passive motor movements were easily elicited from the precentral gyrus, confirming its role as primary motor cortex (M1). Cortical stimulation anterior to M1 (marker 3, see Supplement 1A) resulted in a sudden discontinuation of movement during a continuous flexion-extension task of the left arm and hand; these “negative motor” sites are typically found in non-primary motor cortex (Filevich et al. 2012; Rutten 2015). Guided by tractographic results we stimulated the cortical termination point of the FAT on the premotor cortex (marker 4, Supplements 1A en 2A), and this yielded errors during a picture naming task and stroop-task.

When the resection encroached upon the FAT, as indicated by the surgical guidance system, we started subcortical electrical stimulation using different tasks to assess top-down executive functions: the stroop-task that measures inhibition, and the digit span backward test that measures working memory. Four positive sites were found that were surrounded by many negative sites (Supplements 1B and 2B). Stimulation yielded in an inability to perform the digit span backward test, while performance on the digit span forward test remained normal. Performance on the stroop-task was impaired in six out of ten stimulations. These findings suggest that the right FAT is necessary for working memory, but does not influence recall or attentional capacity and is involved in inhibition. Positive subcortical stimulation sites accurately matched the course of the FAT within FLAIR abnormalities (note that negative sites were not marked nor photographed). A small part of the tumor was therefore left in situ.

### Diagnosis

Oligodendroglioma WHO grade II, codeletion 1p 19q.

### Postoperative cognitive testing

Postoperative screening was conducted at 3 months after surgery, and indicated persisting impairments in verbal memory, motor speed and processing speed. Significant improvements were found for phonemic fluency, inhibition and shifting attention, after correction for practice effects (Rijnen et al. 2018).

## Discussion

In our patient, intraoperative electrical stimulation of the right FAT disrupted executive functions. Positive stimulation results were found along the course of the FAT as indicated by tractography, and were specific in the sense that nearby subcortical sites did not yield abnormal task responses. The dissociation between performance on the digit span forward and backward suggests impairment of executive functions rather than of motor-related functions, given that the motor sequencing requirements are similar for both tasks. Our observations are in line with the neurocognitive literature that suggests involvement of the FAT in networks for executive control (Dick et al. 2019).

A few clinical studies have found evidence for involvement of frontal subcortical regions in cognitive control with electrical stimulation. Puglisi et al. used a stroop test and found interferences during stimulation beneath the right inferior and middle frontal gyrus, and in the periventricular white matter lateral to the caudate nucleus (Puglisi et al. 2019). They linked their positive stimulation sites to inferior fronto-striatal tracts and the anterior thalamic radiation. Nakajima et al. found positive responses during an n-back task they administered during resection of a tumor in the left SMA in two patients (Nakajima et al. 2014). Papagno et al. investigated the cortical and subcortical regions underlying verbal working memory with DES and found distinct areas for item and order storage in frontal and frontoparietal networks (Papagno et al. 2017).

The question that remains to be answered, however, is whether or not the FAT is truly critical for normal cognitive performance, and thus is an ‘eloquent’ structure as seen from a neurosurgical perspective (Kahn et al. 2017). DES is unable to fully answer this question, given its inherent limitations (Mandonnet et al. 2010). DES is a powerful surgical tool due to its high negative predictive value for a targeted structure (i.e., negative stimulation predicts absence of a permanent deficit). However, its positive predictive power, in particular for non-primary functions, is yet unknown and seems limited. There are three main reasons for this. First, DES is non-local, as there is backspreading via the stimulated structure into networks. Second, the ecological validity of these tasks is unknown (i.e., we do not know very well how intraoperative cognitive task performance predicts behavior in the real world). Third, the stimulated region may (eventually) be redundant for the function, because of postoperative functional reorganization or compensation. However, as it is uncommon to resect an area that produces a deficit using DES, long-term clinical outcome of resected positive mapping areas is unknown.

Evidence supporting a critical role of a fiber pathway, such as the FAT, should therefore come from lesion-deficit studies. Selective damage to a tract, however, is rare. Pathological and surgical lesions usually damage larger areas and multiple fiber pathways. In rare cases, though, this information can be highly valuable (Mandonnet et al. 2017; Puglisi et al. 2019). Alternatively, multivariate lesion-symptom mapping can be used to relate focal brain injury to cognitive impairments in groups of patients (Arbula et al. 2017). Another approach is to compare two patient series, one operated with task monitoring and one operated without (Mandonnet et al. 2020). Ideally, effects of surgery should be evaluated in a prospective and randomized design, to account for inherent bias in patient handling, but this will be difficult nowadays due to the common clinical belief that DES is the gold standard for preservation of brain functions in general.

In conclusion, electrical stimulation of the right FAT disrupted working memory and inhibitory functions in a patient with a low-grade glioma. Whether or not this pathway is critically involved in cognitive control networks, or can be compensated for, should be assessed in future studies whereby evidence should come from the combined results of lesion-deficit studies, electrical stimulation studies, and structural and functional imaging studies. Importantly, tasks that target cognitive functions should be carefully introduced in clinical practice to prevent clinically irrelevant responses and too early termination of the resection. We advocate a Bayesian approach, whereby a region is selectively targeted and an a priori hypothesis has been formulated based on existing neuroscientific and clinical literature.

## Supplementary Information

**Supplemental material 1 (A)** Photograph of the exposed part of the right frontal cortex. Dotted white line indicates midline. Markers 3 and 4 indicate positive responses over the supplementary motor area (i.e. medial part of the premotor cortex). **(B)** Markers 5,6,7,8 indicate positive subcortical stimulation sites along the trajectory of the frontal aslant tract. Asterisk denotes site of marker 4. **(C)** Resection cavity. (PNG 8383 kb)

High resolution image (TIFF 2577 kb)

**Supplemental material 2** Screenshots from the surgical navigation system. **(A, B)** FLAIR images indicate right-sided low-grade glioma. Colored lines display the results of tractography: frontal aslant tract (blue), corticospinal tract from central lobe (green), inferior fronto-occipital fasciculus (yellow). Straight blue-yellow line indicates the position of pointing device. Device points to marker 4 in screenshot 2A and to marker 7 in screenshot 2B. **(C)** Postoperative FLAIR and DTI images (three days after surgery) with part of the frontal aslant tract (blue) traversing the remnant of the glioma. (PNG 2685 kb)

High resolution image (TIFF 3300 kb)

## Abbreviations

FAT: frontal aslant tract
DES: direct electrical stimulation
SMA: supplementary motor area
DTI: diffusion tensor imaging
MRI: magnetic resonance imaging
