# Supplementary figures and images for: Executive functional deficits during electrical stimulation of the right frontal aslant tract
Source: Brain Imaging Behav. 2021 Jan 19;15(5):2731–5. doi: 10.1007/s11682-020-00439-8 (PMC8500906; doi:10.1007/s11682-020-00439-8)

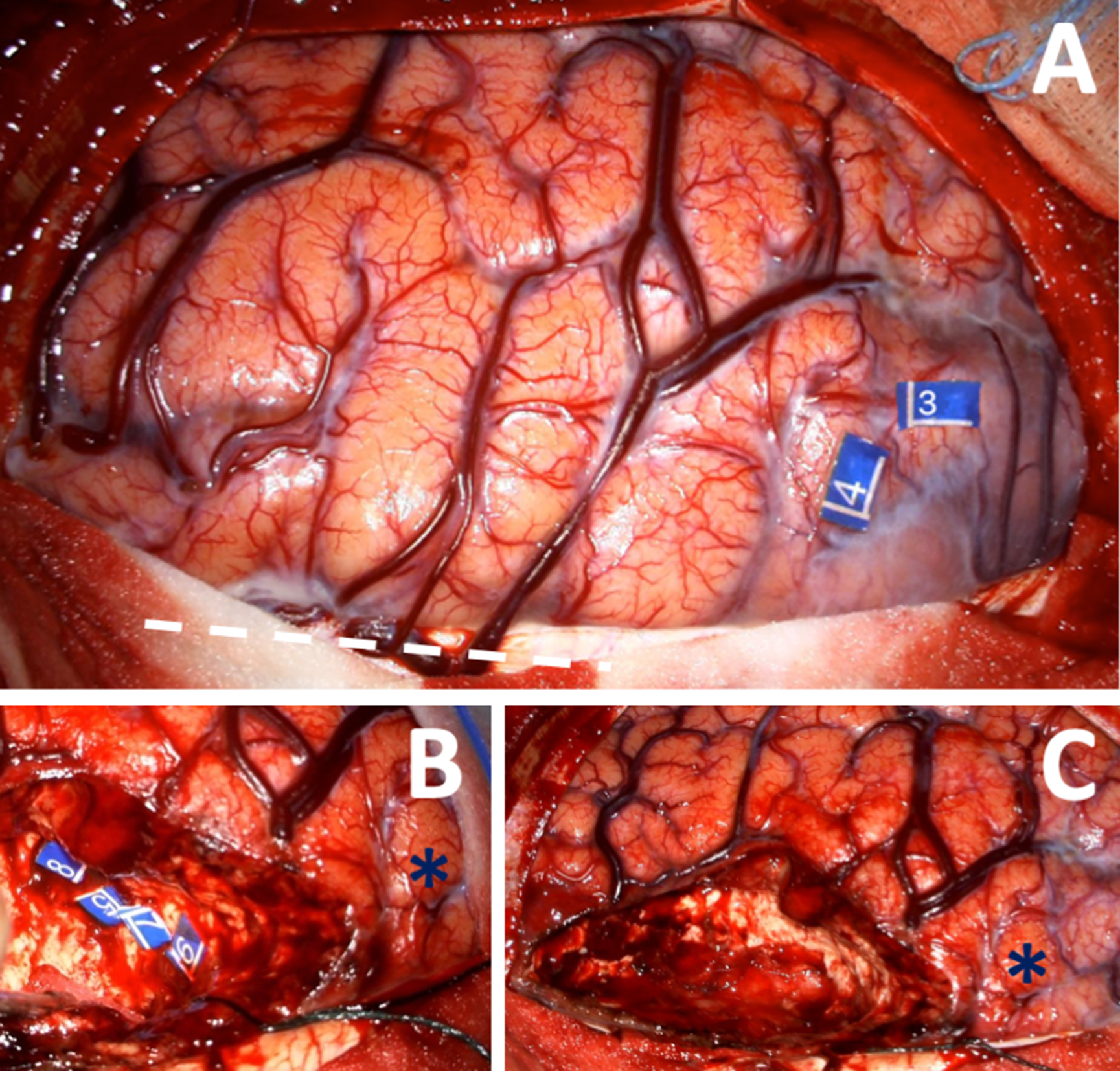

Supplement: Supplementary file 1 — Supplemental material 1 (A) Photograph of the exposed part of the right frontal cortex. Dotted white line indicates midline. Markers 3 and 4 indicate positive responses over the supplementary motor area (i.e. medial part of the premotor cortex). (B) Markers 5,6,7,8 indicate positive subcortical stimulation sites along the trajectory of the frontal aslant tract. Asterisk denotes site of marker 4. (C) Resection cavity. (PNG 8383 kb) [file 11682_2020_439_Fig1_ESM.png]

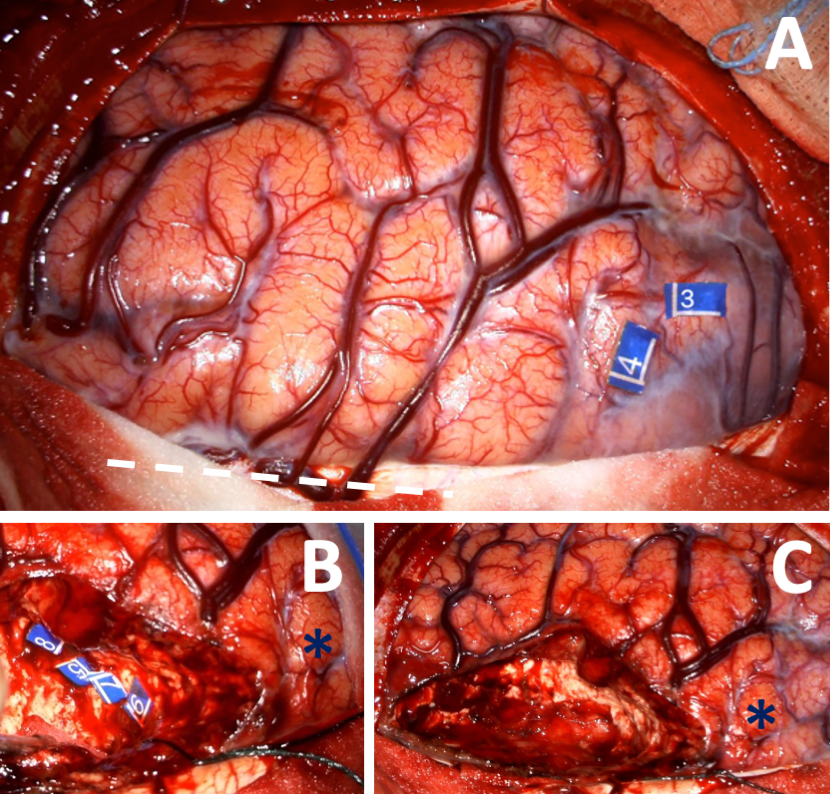

Supplement: Supplementary file 2 — High resolution image (TIFF 2577 kb) [file 11682_2020_439_MOESM1_ESM.tiff]

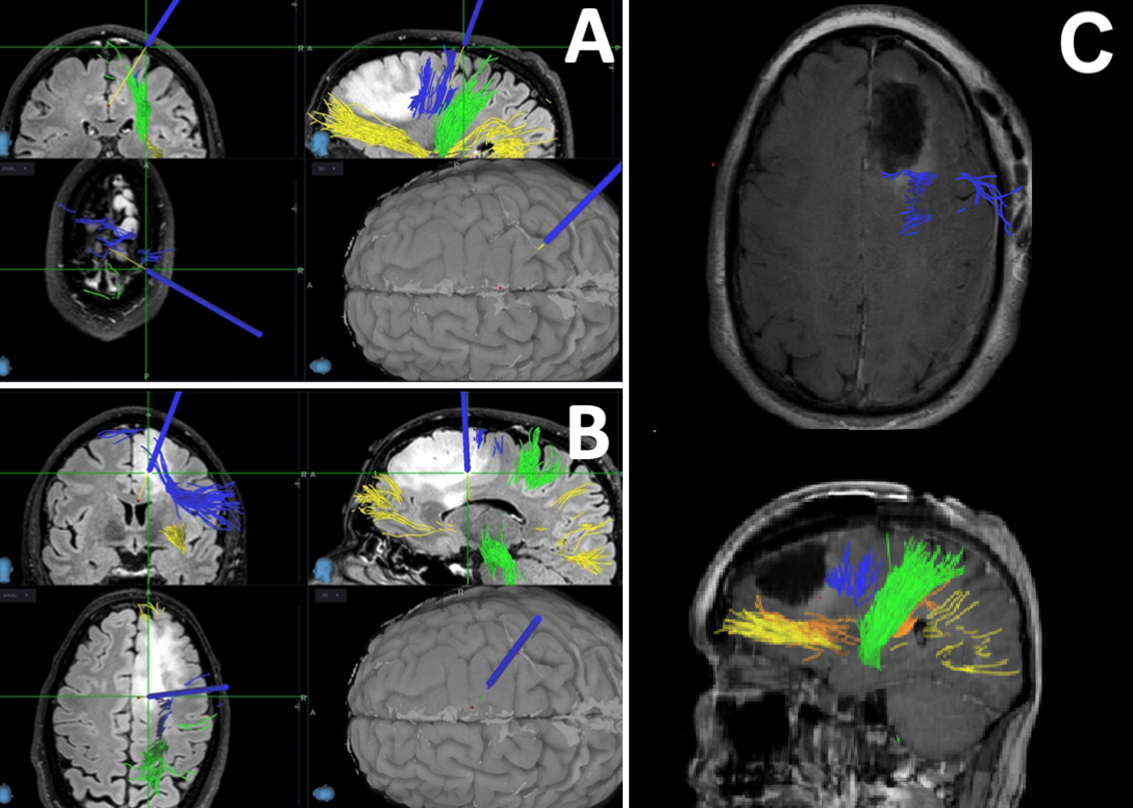

Supplement: Supplementary file 3 — Supplemental material 2 Screenshots from the surgical navigation system. (A, B) FLAIR images indicate right-sided low-grade glioma. Colored lines display the results of tractography: frontal aslant tract (blue), corticospinal tract from central lobe (green), inferior fronto-occipital fasciculus (yellow). Straight blue-yellow line indicates the position of pointing device. Device points to marker 4 in screenshot 2A and to marker 7 in screenshot 2B. (C) Postoperative FLAIR and DTI images (three days after surgery) with part of the frontal aslant tract (blue) traversing the remnant of the glioma. (PNG 2685 kb) [file 11682_2020_439_Fig2_ESM.png]

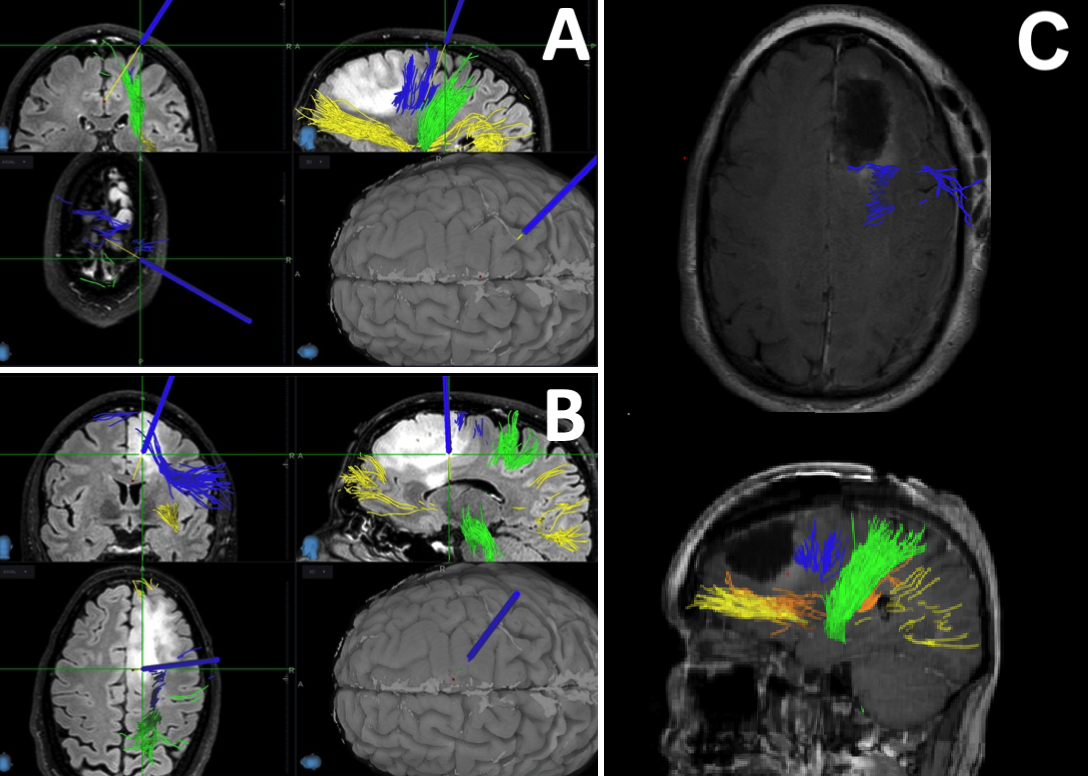

Supplement: Supplementary file 4 — High resolution image (TIFF 3300 kb) [file 11682_2020_439_MOESM2_ESM.tiff]
